# 

**Published:** 1888-10-06

**Authors:** 


### The Hospital

A Weekly Institutional Journal of

### Science, Medicine, Nursing, and Philanthropy.

EDITED BY

HENRY C. BURDETT,

AUTHOR OF "HOSPITALS AND THE STATE"; "PAY HOSPITALS OF THE WORLD"; "COTTAGE HOSPITALS, GENERAL, FEVER, AND CONVALESCENT, WITH FIFTY BEDS AND UNDER: THEIR ORGANISATION, MANAGEMENT, AND WORK," ETC., ETC.

ACTING EDITOR:

GEORGE W. POTTER, M.D.,

AUTHOR OF PAPERS ON "BACTERIOLOGY"; SCARLET-FEVER"; "RHEUMATISM"; ETC., ETC.

INDEX TO VOL. V.

(6th October, 1888, to March 30th, 1889.)

FOR 1888-9.

SURGEON GEN'L'S OFFICE LIBRARY

LONDON:

PUBLISHED BY "THE HOSPITAL" (Limited), 139 and 140, SALISBURY COURT, FLEET STREET, E.C.

WI
H069X

### INDEX TO VOL. V. 1888-9.

Alexandra Hospital for Sick Children, 8
Accommodation for Diphtheria in London, 8
After-Care Association, 9
Annotations, 11, 27, 43, 59, 75, 91, 107, 123, 139, 154, 171, 187, 201, 221, 237, 253, 269, 285, 301, 317, 333, 348, 365, 381, 397, 413
Amusements and Relaxation, 1, 6, 32, 48, 64, 80, 96, 112, 128, 144, 160, 176, 192, 210, 226, 242, 258, 274, 290, 306, 318 338, 354, 370, 386, 402, 418
Asylums, Round About the, 22
Australasian Science Association, 52
Annual Meetings:
—— Salford and Pendleton Royal Hospital, 9
—— Society of Medical Officers of Health, 73
—— Royal Albert Hospital, Devonport, 121
—— Great Northern Central Hospital, 282
—— Charity Organisation Society, 283
—— Italian Hospital, 298
—— Royal Ear Hospital, 298
—— Royal Free Hospital, 314
—— Charing Cross Hospital, 331
Anting Missionary Hospital, Pekin, 73
Alcohol, Effect of, on Morality and Health, 106
Addenbrooke's Hospital, Cambridge, 114
A Plaintive Cry, 123
Alcoholic Paralysis, 124
Are we a Nation of Liars? 129
Ancient Greeks, Hospitals Amongst the, 148
Animals, Experiments on, 150
Ancient and Modern Metropolitan Hospitals, 174, 214, 239, 246
Alcoholic Diseases, 216
All Saints' Convalescent Home, Eastbourne, and *Truth*, 218
Adelaide Hospital, Dublin, 225
Alehouse, the, and the Hospital, 247
Adulterated Milk, 253
Appetite, Coaxing the, 269
After Dinner Walk a Mile, 301
Abstainers, are they Long Lived?, 317
Australasia, the Intercolonial Medical Congress of, 345, 358
Accidental Poisoning of Children, 349
Army Surgeon, an Old, 352
Ambulance System, the London, 378, 403
Alabama Lunatic Asylum, 382
Aberdeen Infirmary, Extension of, 395

Burdett, Mr. H. C., presents Stained-Glass Window to Seamen's Hospital, 41
Bristol Royal Infirmary, 41
Bath, the Only Safe, 43
Blood, the, 60, 109, 143, 167
Brine Baths for Rheumatism, 71
Brompton Hospital, 73, 219
Bequests to Hospitals, 73, 95, 362, 363
Bolton Infirmary, 98
British Home for Incurables, 104
Beddington Female Orphan Asylum, 120
Broadmoor Criminal Lunatic Asylum, Alleged Jobbery at, 121
Bristol General Hospital, 136
Berlin Children's Hospital and the Empress Frederick, 136
Belfast Royal Hospital, 137, 362,
Bread or a Stone, 171
Boycotting Sir Morell Mackenzie, 171
Boy Ruffianism in London, 187
Brain Injury and Bad Temper, 216
Bad Temper and Brain Injury, 216
Babies, 217, 238, 254, 265, 281, 297
Birmingham Workhouse Infirmary and the *Lancet*, 235
Birmingham, New Infirmary for, 250, 267
—— General Hospital, 136, 411
"Bleeding" Revived, 255
Blackheath Cottage Hospital, 257
Brute, a Human, 280
Brighton Hospital for Women, 298
—— Charities, 322
Boarding Out of Children, 331
Breakdown of the Hospital System, 341, 357, 393, 404
Bradford Infirmary, 346
Belfast Consumption Hospital, 347
Baby Farming Superseded, 329
Back-to-Back Houses, Unhealthiness of, 379
Brain, the, as a Working Tool, 381
Bootle Borough Hospital, Alleged Inhumanity at, 381, 413
Bath and her Waters, 382
Bristol General Hospital, 395

Chemists and Doctors, 5, 91
Cocoa, 6
Clarke, Sir Andrew, on Examinations, 11
Children's Corner, 16, 32
Charities, the Rating of, 17
Children's Cast-off Clothing for Hospitals, 41
Children, Society for Prevention of Cruelty to, 65, 330
Childhood, the Empire of, 65
Cigarettes, the *Lancet* on, 73
Consumption Hospital, Brompton, 73, 219
City Magnates and their Work, 81
Consumption, the Treatment of, by a Berlin Physician, 84
Copenhagen and the Baltic, 87
Cardiff Infirmary, appointment of Ophthalmic Surgeon, 88
Cinchona, the Cultivation of, 90
Cronstadt and St. Petersburg from the Sea, 103
Cremation Society, 104
Cromer Cottage Hospital, 104
Cork South Infirmary, 114
Competitive Examinations, 123
Charing Cross and Other Hospitals, by the Earl of Derby, 131, 135
Children's Hospital, Dublin, 137
—— Berlin, and the Empress Frederick, 136
—— Paddington, 234
Common Humanity in Lancashire, 381
Chemists and their Work, 165
Charity Appeals, 168
Cancer Cases, Isolation of, 169
—— A Hundred Years Ago, 329
Christmas Entertainments, 184, 218, 219, 234, 235, 250, 251, 266, 267, 282, 283, 298
—— Carol, a, in a Minor Key, 195
Congleton Cottage Hospital, 234
Coughing in Church, 237
Convalescent Homes opened in 1888, 250
Consumption: Is it Contagious? 260
Central London Throat Hospital, 267
Cremation, 269
Charity, a Land of, 278
—— Organisation Society, 283, 378
Cure, the Universal, 285
Chalmers' Hospital, 298
Criticism of Cash, 307
City of London Truss Society, 314
Cleanliness, the Cult of, 319
Child Murder, Premiums on, 323, 330
City of London Lying-in Hospital, 330
Charing Cross Hospital, Annual Court, 331
Colonial Congress, a, 345, 358
Croydon Provident Dispensary, 347
Cork Fever Hospital, 347
Child Suicides, 349
Coal Mine, Visit to a, with Miners, 361, 377
Conveyance of Injured Persons to the Hospitals, 378
Court, from the, to the Hospitals, 381, 406
China, Medical Practice in, 411

Doctor's Pulpit:
The Seven Ages of Man—
—— The Infant, 3
—— The Schoolboy, 21
—— The Lover, 35
—— The Soldier, 51
—— The Justice, 69
—— The Lean and Slippered Pantaloon, 85
—— Sans Everything, 102
Present Day Problems—
—— The Question of Population, 115
—— At Hand-Grips with Destiny, 133
—— Wasters, 149
—— Social Parasites, 163
—— Cures for do. 179
All Sorts and Conditions of Women—
—— Preliminary Considerations, 213
—— Four Created Women, 229
—— The Intellectual Woman, 245
—— The Mother of Children, 261
—— The Best Wife, 277
—— The Worst Wife, 293
—— Are Old Maids Failures? 309
—— When Should Girls Marry? 325
—— The High Priestess of the Home, 343
Donegal County Infirmary, 5
Doctors and Chemists, 5, 91
Diphtheria, 222
—— Accommodation for, in London, 8, 49, 104
Dover, New Hospital for, 40
Dangerous Ointments, 75
Discourse to Women on Medical Subjects, 79
Drowning, Sensations of, 90
Dundee Royal Infirmary, 98, 140, 148
Doncaster Infirmary, 104
Derbyshire General Infirmary, 104
Derby, the Earl of, on Hospitals, 131
Doctors' Fees, 139
Dublin Children's Hospital, Silver Fete, 153
Dispensaries, Provident and Free, 169
Dental Hospital of London, 184
Death, Absolute Signs of, 218
Desperate Diseases and Desperate Remedies, 253
Dried-up Pensioners, 301
Durham County Hospital, 314
Diphtheria Microbe, the, 314
Deaf and Dumb, Teaching the, 344
Dewsbury Hospital, Some Cases Treated at, 347
Devon and Exeter Hospital, 363
Doctors' Disabilities, 371
Doctor's Pulpit—
—— Man and his Enemies, 359
—— Inherited Taints, 373
—— Birth and Babyhood, 389
—— Mental Excitement, 405
Doctors, the Compulsory Elevation of, 387
Dental Hospital of London, 394

East End Murders, 8, 88, 122, 137
Everybody's Page, 10, 26, 42, 58, 74, 90, 106, 122, 138, 154, 170, 186, 202, 220, 236, 252, 268, 284, 300, 316, 332, 348, 364, 380, 396, 412
Examiners Examined, 11
Edison's Phonograph at St. Mary's Hospital, 18
Eyes, their Capacity for Reproduction, 58
Empire of Childhood, the, 65
Enfield Cottage Hospital, 73
Editor's Letter Box, 95, 101, 175, 215, 274, 292, 338, 342, 393
Experiments on Animals, 150
"Encyclopædia Britannica," 169
Eating House Trick, the, 221
Eyes and Sight, 227
Enlargements of Hospitals in 1888, 250
Egypt, a Winter in, 276
East Wind Liver, 317
Essex and Colchester Hospital, 346

Friendly Societies, 8, 25
False Impressions, by Caroline Fothergill, 13, 29, 45, 63, 77, 93, 110, 126, 141, 157, 173, 188, 223, 240, 256, 271, 287, 304, 321, 334, 351, 367, 383, 399, 415
Fallen Women, 27
"Frederick the Noble," 37
Fogs and Town Smoke, 53
Fever and Fever Hospitals, 57
Falkirk, a Cottage Hospital for, 57
Fish as an article of Diet, 57
Fever Hospital, the London, 67
French Hospital and Dispensary, 70
False Doctoring, 91
Flock, the, or the Fleece, 91
Fright and its Consequences, 124
Faversham Cottage Hospital, 136
First Aid to the Injured, 182
Faith Healing, 184
Funeral Wreaths, 187
Flower Girls and *The Times*, 234
Football Ear, 280
—— Brutalities, 317
Fat, 302, 397
Fiji and the Coral Islands, 326
French Hospital and Dispensary, 331
Football, and Hospital Collections, 363
Flowers at the Queen's Drawing Rooms, 381, 406
Fancied Pains,
Friendly Societies, Financial Position of, 417

German Hospital, 5
Griffith Bequests, the, 73
German Taptoo, a, 82
Gladstone, the Right Hon. W. E., and Longevity, 97
General Hospital, Birmingham, Sanitation of, 136, 411
Greeks, Hospitals amongst the Ancient, 146
Glasgow Western Infirmary, 152
Givers and Giving, 178
Germs, Something about, 181
German Hospital, 234
Great Northern Central Hospital, 282
Give it a Fair Trial, 291
Glasgow Royal Infirmary, 299
Grosvenor Hospital for Women and Children, 331, 389
Growing Fat, 397

Halifax Infirmary, 379
Horseflesh to the Fore, 365
Horse, the, and his Murderers, 365
Hospital System, Breakdown of the, 341, 357, 404
Hints for the Sick-Room, 328, 385, 398, 414
Health in our Homes, 311
Horse, Extract of, 301, 365
Holloway Sanatorium at Egham, 296
Health, the High Roads to, 295
Health Society, the National, 251
Hants County Asylum, 225
Hospitals Association, Meetings of, 8, 23, 47, 88, 152, 378
—— and the National Pension Fund, 23, 47
—— and Sixteen Years of Hospital Sunday, 81, 88, 116, 117
—— and Hospital Extravagance, 152, 189, 194
—— and London Ambulance System, 410
—— and Patients' Contributions to Hospitals, 288
—— Entertainment in Aid of, 394
Hospital Physicians and Surgeons and Hospital Appointments, 8

Hospital Finance and Economy, 19, 83
Hospital Smack for North Sea Fishing Grounds, 24
Homœopathy at Birmingham, 25
*Hospital*, the, and the *Lancet*, 39
——— the Circulation of, 152
Hospital Nurse and Children's Cast-off Clothing, 41
Hospital Saturday Fund, Metropolitan, 8, 137, 200, 283, 345
——— Richmond, 72
Hull Royal Infirmary, 70, 120
Homœopathic Hospital, the London, 89
Hospitals and Socialism, 125
Hot Bottles in Bed, 139
Hand-Fed Children, 150
Hospital Extravagance and Expenditure, 152, 189, 194
Hospital for Sick Children, 185
Hospitals at Home, 201
Hospital Sunday, Sixteen Years of, 81, 88, 99, 116, 117, 156
Hospital Sunday, Metropolitan, 283
——— a New Departure by, 137, 168
——— Birmingham, 9
——— Salisbury, 40
——— Devonport, 72
——— Worcester, 72
——— Ballymena, 105
——— Romford, 135
——— Norwich, 282
——— Inverness, 282
——— Romford, 414
Highland Laird, the Family of a, 390
Hastings Hospital, 411

In and Out Among the Hospitals, 4, 70, 98, 114, 225
Isle of Man General Hospital, 4
India, Military Graveyards in, 12, 368, 375, 400, 407
*Illustrated Medical News*, 16
Incompatible Mixture, an, 91
Infectious Hospitals, Effects on Value of Adjacent Property, 104
Impressions of St. Petersburg, 119, 135
Isle of Wight Infirmary, 120
Insane, Glances at the, in other Lands, 134, 164, 231, 262, 353, 369, 382
Infectious Hospital for Sutton, 136
Indian National Association for Supplying Female Medical Aid, 147
Injured, First Aid to the, 182
In Spare Moments, 244
Illustrated Papers and the Hospitals, 250
Infectious Diseases, Hospitals for, 253, 295
Jubilee Hospitals, 40, 282, 346, 410
Insane, Holloway Sanatorium for, 296
Italian Hospital, 298, 363
Insurance of Children, 323, 330, 334
Insomnia, 350

Knock-Knees, 20

King's College Hospital Revisited, 50

——— Christmas at, 283

——— 362

Long Case, a, 20
*Lancet* and the *Hospital*, 39
——— on Cigarettes, 73
Leicester, New Children's Hospitals for, 40
Lunatic Asylums and Visiting Physicians, 41
Leeds as a Leader, 43
Local Government Board and Isolation of Diphtheria, 49
Leicester Infirmary, Financial State of, 56, 89
Lady Guardians, 59
London Fever Hospital, Story of, 67, 331
Lover's Choice, a, 75
Lunatic, a Dangerous, 84
London Homœopathic Hospital, 89
Little Men, 92
Longevity, an Object Lesson on, 97
——— and Temperance, 317
Lord Mayor's Procession, 107
Lunatic, a Definition of a, 107
Ladies, Training of, at National Dental Hospital, 120
Liars, are we a Nation of, 129
Leprosy, Something about, 145
Lockjaw, Cases of, 168
Lancaster, New Infirmary for, 184
Let Him Die, 201
London Hospitals and Other Charities, 203, 218
——— Christmas at the, 219
——— Quinquennial Appeal, 251
Leeds General Infirmary, 225, 378
*Lancet*, the, and Birmingham Workhouse Infirmary, 235
*Lancet*, the, and the Pension Fund, 257
Liverpool Royal Infirmary, 299
Literature in Plague Time, 310, 324, 340
Lepers and Father Damien, 331, 346
Leamington Home for Incurables, 362
Liverpool Dispensaries, 363
Leprosy, a Case of, in Dublin, 394
London to be Ambulanced, 378, 403
London in 1828, 406
Lancashire, Common Humanity in, 381, 418

Murders in the East End, 8, 88, 136
Montrose Royal Lunatic Asylum, 9
Mackenzie, Sir Morell's, Book, 11
——— his Defence, 43, 171
Military Graveyards and Epitaphs in India, 12, 368, 375, 400, 407
Malingering, 15
Manchester, a New General Hospital for, 24
Mutual Friendly Aid Society, 24
Merthyr, Opening of Hospital, 25
Myopia, 26, 42
Malthus and the Vicar, 27
Menthol, the Use of in Pulmonary Phthisis, 42
Murder and Penny Dreadfuls, 59
Macaulay on Smallpox, 68
Milk as a Medicine, 74
M'Kellar Bequests, the, 95
Mercer's Hospital, Dublin, 105
Medical Monopolies and General Practitioners, 113
Meat Extracts, 138
Metropolitan Hospital, 169, 298
Metropolitan Hospitals, their Work and Financial Position, 174, 214, 239, 246
Metropolitan Asylums Board and Measles, 185, 298
Metropolitan Asylums Board and Measles, 185, 267
Manchester, High Death-rate at, 218
Measles, 185, 230, 298
Moscow, 233
Milk, Adulteration of, 253
Man and Medicine, 260
Medical Education, Relation of Hospitals to, 279
Mendicity Society, the, 299
Medical Practitioners, Prospects of, 333
Medical Aids and Appliances, 344
Manchester Provident Dispensaries, 346
Modern Neros and their Fiddles, 349
Man and his Enemies, 359, 373, 389
Middlesex Hospital, 362
March Dust, 365
Mental Excitement, 405

Newcastle Royal Infirmary, 330
Norway and the Land of the Midnight Sun, 303, 318
Navy, Work in the, 169
Notes and News, 8, 24, 40, 56, 72, 88, 104, 120, 152, 168, 184, 200, 218, 234, 250, 266, 282, 298, 314, 330, 346, 362, 378, 394, 410
National Pension Fund for Nurses, 8, 16, 23, 34, 46, 57, 235, 266
——— and the Nottingham and Notts Nursing Association, 16
——— and the *Lancet*, 237
New Remedies and Appliances, 111
New Cures, 107
Nottingham and Notts Nursing Association, 16
North Sea Fishing Grounds, Hospital Ship for, 24
Norwich and Norfolk Hospital, 56
Nitrite of Amyl as a Weapon of Warfare, 90
National Association of Medical Aid, 105
National Dental Hospital, Ladies Trained at, 120
Nurses, a New Opening for, 187
——— A Register for, 211
National Hospital for Paralysed and Epileptic, 219
Nurses, the Sweating of, 221
Nerve Centres, 228
North Staffordshire Infirmary, 235
New Hospitals opened in 1888, 250
National Orthopœdic Hospital, 267
Nervous Diseases, West-end Hospital for, 267
New Hospital for Women, 330
New Surgical Appliances, 337
New Brunswick Lunatic Asylum, 353
Nottingham General Hospital, 376
National Thrift, 388

Opium, the Use of, in India, 411
Other People's Money, 11, 38
Odd Fellows', A. O. of, Convalescent Scheme, 52
Overwork, 161

Philanthropist's Vade Mecum, the, 203, 218
Patti, Madame, Charity Concerts, 120
Present Day Problems, 115, 133, 149, 163, 179
Physiology and Murder, 1
Preparations, New, 6
Pleasure Cruises Available, 7
Physicians and Hospital Appointments, 8
Physicians, College of, and Sir Morell Mackenzie, 11
Phonograph, the, at St. Mary's Hospital, 18
Pension Fund, the National, 8, 16, 23, 34, 46, 57, 235, 237, 266
Pleasure Yachts, Deficiencies of, 44
Providence, Free Hospital, St. Helens, 57
Penny Dreadfuls and Murder, 59
Physiology and its Uses:
——— The Blood, 60, 109, 143, 167
——— Fat, 302
Physical Culture of Women, 153
Processions or Puddings, 107
Poetry, 51, 241, 361
Prevention of Cruelty to Children, 65
Poor Law System and Medical Relief, 72
Provincial Hospitals, the Financial Position of, 83
Provident Dispensary, How to Establish a, 104
Physicians, a Distinctive Dress for, 106
Queen's Jubilee and the Hospitals, 9, 40
Pennsylvania Asylums, 231
Paddington Children's Hospital, 234
Poplar Hospital, 235
Penny a Week Working Men's Collection, 234, 283
Power of the Penny, the, 243
Phthisis, Treatment of at High Altitudes, 256
Patients' Contributions to Hospitals, 263, 283
Plymouth Royal Eye Infirmary, 283
Physicians' Whims or Women's Faults, 285
Pap, What is it? 285
Provident System, the, 292
Perth City and County Infirmary, 315
Pears' Soap, 319
Police Matrons, 339, 374
Portsmouth, &c., Royal Hospital, 379
Perfect Report, a, 379
Pyscho-Therapeutics, 391
Punishment of Boys, 397
Portobello, Proposed Hospital for Infectious Diseases, 413
Posture, Importance of, 414

Queen Charlotte's Hospital and the Princess Christian, 266
Queen's, the, Visits to Hospitals, 315

Richmond Friendly Societies, 8
Royal Lunatic Asylum, Montrose, 9
Rotunda Hospital, Dublin, 10
Rating of Charities, 17
Round About the Asylums, 22
Reviews, 31, 37, 61, 86, 159, 180, 247, 312, 356, 374, 390
——— "Frederick the Noble," 37
——— "Self-Help a Hundred Years Ago," 86
——— "Olfactics and the Physical Senses," 108
——— "Massage," 118
——— "Heroes of Every-day Life," 159
——— "Hydropathy and Health," 295, 391
——— "Psycho-Therapeutics," 391
Rheumatism, Brine Baths for, 71
Religious Consolation for Patients in Infectious Hospitals, 91
Religious Consolation for Patients in Stockport Infirmary, 105
Royal Albert Hospital, 121
Russian Palaces and Churches, 151, 172
Russian Royal Carriages, 183
Ross, Major Alexander, M.P., Death of, 152
Ring Out the Old, 193
Register for Nurses: Is one Desirable? 211
Royal Albert Orphan Asylum, 212
Russian Dinners, Hotels, &c., 249
Rod or Cane, 276
Royal Ear Hospital, 298
Royal Free Hospital, 314, 410
Royal Berkshire Hospital, 378
Retribution, 397

Seven Ages of Man, the, 3, 21, 35, 51, 69, 85, 102
Sussex County Hospital, 4
Surgeons and Hospital Appointments, 8
Sims Reeves, Life of, 8
Salford and Pendleton Royal Hospital, 9, 57, 315, 346
Serious Case against a Hospital, Alleged, 14
St. Mary's Hospital Conversazione, 18
——— Edison's Phonograph at, 11
——— Christmas at, 250, 267
St. Mary's Hospital, Manchester, 410
Surrey County Hospital, 24
Sea-Sickness: Is it a Myth? 28
Shaftesbury Memorial, the, 31
Sectarianism at London Hospitals, 33
Soldiers and Teetotalism, 38
St. Albans, New Jubilee Hospital for, 40
Sunnyside Asylum, 40
Swindon Hospital, 40
Seamen's Hospital, Greenwich, Stained Glass Window Presented, 41
——— Christmas at, 219
Scrofula, Asylum of House of Industry, Dublin, 42
Stockton-on-Tees Hospital, 56
St. Helens Providence Free Hospital, 57
Students' Column, 62, 70, 94, 130, 166, 178, 224, 310, 337
Scotch Asylums, 66
Smallpox, Macaulay on, 68
Sea Travel, Advantage of, 76
Scraps and Gleanings, 80, 96, 112, 128, 144, 160, 176, 192, 226, 242, 258, 274, 290, 306, 318, 338, 354, 370, 386, 402, 418
Sixteen Years of the Hospital Sunday Fund, 81, 88, 99, 116, 117, 156
Stockport Infirmary and Religious Ministrations to Patients, 105
Smell, How to Cultivate the Sense of, 108
Shannon File, the, 111
St. Petersburg, Impressions of, 119, 135
——— to Stockholm by Sea, 270
South Africa as a Health Resort, 121
Salford, Infectious Hospital for, 121
Socialists and Hospitals, 125
Special Hospitals, the Case for, 132
Sutton, Infectious Hospital for, 136
Sanitation of General Hospital, Birmingham, 136
Silver Fete at Children's Hospital, Harcourt-street, 136
St. John's Ambulance Association, 153
Silver Fete at Dublin Children's Hospital, 153
Shop Assistants, Slaughter of, 155
Shop, the, and the State, 155
State, the, and the Shop, 155
Saccharine: Is it Injurious to Health? 162, 308
South London District Nursing Association, 201
Surgical Aid Society, 219
Sweating System and Nurses, 221

122919

Suicide and Insanity, 221
Sheffield General Infirmary, **225**
Sunderland Infirmary, 225
Samaritan Free Hospital, 235
St. Paul's, Cray, Cottage Hospital, **251**
St. George's Hospital, Christmas at, **266**

Smoking, 269
Sensationalism in Public Performances, **273**
Sweden and Stockholm, 286
Short Sight and its Causes, 294
Surgery and Therapeutics, 308
Spine, Curves of the, 308
Sidmouth Cottage Hospital, **315**
Sailors' Homes, 327
Seamen, a Word for our, 327
Surgical Appliances, New, 337
Sea-Sickness, the Pathology of, **344**
Swedish Mechanical Exercise, 344
Skye, Isle of, Proposed Hospital for, **346**
Samaritan Society, 347
Suicide of Children, 349
Sleeplessness, 350
Students' Reckoning Day, the, **355**
Stirpiculture, 372
Southport Infirmary, 378
Secretaries and Appeals, 378
Sleep and Suggestion, Treatment by, **391**
Shampooing *versus* Massage, 392
Suffolk General Hospital, 394
Science and Surgery, 409
Skin, the, as an Indicator, 409
Sea Shell and Scrapbook Mission, **410**
St. John's Hospital Scandal, **412**

**Town Smoke and Fogs, 53**
Tyrannies of the Workshop, 59
Tricycles and Medical Men, 74
Tynemouth Infirmary, Building Operations, **105**
——— Story of the, **137**
Temperance at the East End, **122**
Tea, 139
*Truth* Exhibition of Toys, **185**
Tinned Meats, 216
Type Writing, 244
Temperature Taking, Hints for, **328**
Teachers, Medical, 355
Treatment by Sleep and Suggestion, **391**

Unemployed and the Lord Mayor, **171**
Universal Cure, the, 285

Vacation, Voyages in, 7, 28, 44, 55, 76, 87, 103, 119, 135, 151, 172, 183, 233, 249, 270, 286, 303, 313
Ventilation of Rotunda Hospital, 10
Voice, Cultivation of, 10
Vaccination : Is it a Failure? 36, 169
Visiting Physicians to Lunatic Asylums, **41**
Vegetarianism and Cancer, 124, 152
Voluntary Hospitals, a Plea for our, **194**
Vegetarianism, 218
Vicarious Digestion, **237**
Vermont Asylum, 262
Victoria, New South Wales, Lunatic Asylums, 369

Within the Wards, 2, 20, 84, **124**, **150**, **216**, 228, 255, 280, 329, 366, 409
Women, a Defender of, 27
Women, Fallen, 27
**Working Men and Hospitals, 41**
Wolverhampton Eye Infirmary, **56**
Workshop, Tyrannies of the, 59
Women and their Victims, 75, 89, 140, 175
Women, Discourse to, on Medical Subjects, **79**
Whiskey and Snake Poison, **90**
Waist Belts and Stays, 106
What is a Lunatic? 107
West Cornwall Miners' Hospital, Redruth, **114**
Worneford Hospital, Leamington, Enlargement of, 120
Women and Medicine, 122
Women : Are they Deteriorating? **123**
Wet Feet, 139
Women of India, Female Medical Aid for, **147**
Women, Physical Culture of, **153**
Washing Bills, 155
Woodford Girls' Home, 169
——— Convalescent Home, **266**
Warning, a Painful, 201
Women, all Sorts and Conditions of, **218**, **229**, **245**, 261, 277, 293, 309, 325, 343
Working Men's Penny a Week Collection, **243**, **283**
Wry Neck, 280
Weymouth Royal Hospital, 299
Women's Help for Woman, 339
Wimborne Jubilee Hospital, 346
Wound Dressing, Recent Advances in, **366**
Wirral Homœopathic Hospital, **379**
Watcher, the, 406
West Heath Sanatorium, 411
**Working Men and Hospitals, 411**

Yellow Fever, 169
Year, Ring Out the Old, 193
Yarmouth Children's Convalescent Home, **314**
**York County Hospital, 394**

### THE NURSING MIRROR.

Alfred Hospital, Melbourne, xi, xv
Australia, Notes from, vi, xxxviii, l, lxx, lxxxvii, xc
American Woman's Work, an, ix
Army Nursing, xix, lxv
Amusement for Nurses, xxxvii
Alexandria, Nurses at, xlix
Abbreviations, Useful, lxxv
Anxious Nurses, lxxxix
Asylum Attendants, ci
Ashton District Infirmary Nursing Home, lxxvii

British Nurses' Association, xi, xiii, xix, xxxiii, xli, xlii, xlv, lxiii, lxiv, lxix
Bromhead, Mrs., a Memorial to, xiii
Birmingham Infirmary, xvii
Born Nurses, xvii
Babies, Inquests on, xxiv
Belfast Nurses' Home, xli
Bristol Nurses' Home, liii
Beaford Provident Dispensary, liii
Boston Cottage Hospital, lvii
Bolton, a Nursing Association for, lxxxi
Brighton, Sussex, County Hospital, Accident at, lxxvii

Children's Hospital, Great Ormond-street, l
Carelessness by Nurses, xxv
Charing Cross Hospital, xxix, xxxiii
Chichester Infirmary, Nursing at, xxix, xxxiii
Continental Hospitals, xli
Certificates, Nurses', xli, lxxxvii
Conversaziones, xlv
Christmas Entertainments, xliii, lix, lxix
Croydon Union Infirmary, liii
Christmas Holiday, a, lix, lxvii, lxxi
Children and Nurses, lxiv
Chester Infirmary, lxxiii
Case Number Nine, lxxiv
Common Abbreviations, lxxv
Combination of Private Nurses, lxxii, lxxxiv, lxxxv, lxxxvii, xcii
Christ's Hospital, Sherburn, xcix

District Nurses for Surrey, i, xlv
Devon and Exeter Hospital, x
Death from Misadventure, ix, lxxvii
Disorganised Infirmary, a, liii
Druggists, Women as, lv
Durham County Hospital, xcv

Everybody's Opinion, vii, xi, xv, xix, xxiii, xxxi, xxxv, xxxix, xliii, xlviii, liii, lvi, lx, lxii, lxxvi, lxxviii, lxxix, lxxxiii, lxxxvii, xcii, xcvi, c, ciii

"Extras" for Patients, xlix

"For Queen and Country," ii

French Sisters of Charity, xxi

Fitzroy House, Home Hospital, xiii

Germany, the Empress of, as Nurse, xiii
Game and Fruit, Presents of, for Nurses, xxxvii
Girl Nurses, xxxvii, xxxix
Grosvenor Gallery Conversazione, xiii
Glasgow Royal Infirmary, lvii, lxi

Human Body, the General Structure of, xiv
Homœopathic Nurses, xxv
Hospitals Association, the, xxxiii
Home Hospital, the, xlii
Hospital Extravagance, xlix
Hospital, a Month in, lxii
Heroism, lxxxix, ci
Hampshire Nurses' Institute, ci

Inquests on Babies, xxiv
Insane, Nursing the, xcv, ciii
Insurance for Women, xcviii

Jamaica, a Letter from, lxxix, lxxxiii
Jinny's Twin, xxx
Jottings, xlviii
Jewish Nurses, lxxxv, lxxxvii, xciii

Loquacity, xxii
Lady Nurses, xxxiii, xciii, c
Lady Guide Association, xliv
Lectures to Infirmary Nurses, xlix, lv
London Hospital Conversazione, xlix
Lady Guardians, lviii
Leeds Trained Nurses' Institution, lxxxi
Little One's Mite, the, cii

Massage, i, xix, xliii, lxix
Midwives, Registration of, v, xvii
Matron's Duties, a, x
Mildmay Deaconesses, xxv, xxxv, lxix
Manchester and Salford Sick Poor Nursing Institution, xlvi
Melbourne Benevolent Asylum, l
Midland Counties Training Institution, lxxxv
Medical Missionaries, xci
Medical Press, the, and Nurses, ci

Nursing Journals and Amateur Editors, xcviii
Nurses and Advertisements, xlv
Nurses' Uniform at Great Ormond-street Children's Hospital, i
*Nursing Record* and the National Pension Fund, iii
Nursing, Handbooks on, v
Nurses and Religion, v, vii, xxi
Netley Hospital, Nursing at, ix
National Pension Fund, ix, xxiii, xliii, xlv, li, lvi, lix, lxvi, lxxiii, lxxviii, lxxxi, xciii, c
Nurses' Association, the British, xi, xiii, xix, xxxiii, xlii, xlv, lxiii, lxiv, lxix
Nurses, Presents to, xvii, xxiii, xxvii, xxix, xxxi, xxxv, xxxix
Nursing, one Way of, xxx, xxxiv
Nurses, Rules for, at Taunton, xxxiv
Nursing Lessons in Schools, xxxvii
Nurses' Grievances, xlix
Nurses, Registration of, liv
Novel Nurses, lvii
Night Nursing, lxv
Nurses as Stewardesses, lxxv, lxxvi, lxxx, lxxxiv
Nurses on their Travels, lxxxvi, cii
Nurse Eyre's Mistake, xc
Nursing Sisterhoods, xciv

Original Articles, x, xxii, xxvi, xxx, xxxiv, xlvi, lviii, lxii, lxvi, lxxiv, lxxxii

Our Daily Bread, xxxvii

Pension Fund, the National, ix, xxiii, xliii, xlv, li, lvi, lix, lxvi, lxxiii, lxxviii, lxxxi, xciii, c
Physiology, a Popular Account of, xiv
Probationer, Letters from a, xviii, l
Presents and Nurses, xvii, xxiii, xxvii, xxix, xxxi, xxxv, xxxix
Poetry, xxxii
*Punch* on a Nurse's Position, xxxvii
Presentations, xl, xlvii, lvi, xcvi, c
Provincial Items, xli
Private Nursing, xlii, lv
Philanthropic Sweating, lxix, lvi, lx
Probationers, Discontented, liii, lxi
Paddington District Nursing Association, lxv
Paris, News from, lxxxi
Politeness, lxxxii
Physical Strain of Nursing, lxxxix

Religion and Nursing, v, vii, xxi, lvii, xciv
Royal Free Hospital, xviii, xxxvii
Royal Southern Hospital, xvii
Rich, Sick Nursing among the, xxvi
Reviews, xxviii, xxxvi, xliv, lii, lxxxiii, lxxxviii
Rules for Nurses, xxxiv
Redruth Women's Hospital, xxxviii
Restaurant for Women, xli
Rest and Exercise, lii, lx, lxxvi
Register for Nurses : Is one Desirable? liv
Royal Berkshire Hospital, Nurses, xciii

Sunderland, a Nurses' Home for, v
Sydney Home and Training School, xiii
Shut-in Society, the, xlvi
St. John's House Nursing Association, lxi
St. Bartholomew's Hospital Entertainment, lxiii
Stewardesses, Nurses as, lxxv, lxxvi, lxxx, lxxxiv, xcvii
South London District Nurses' Association, lxxxv
Starting a District Nurse, lxxxvi
St. John the Divine Nursing Institution, xciv, xcvii
Sussex County Hospital, an Accident at, lxxvii

Taunton Nursing Institution, xviii, xxxiv
Trained or Not? xxix, xxxiii, xxxix
Teething Customs, Curious, xlvi
Town Nursing, xlvi
Truthfulness, Scientific Aspects of, lxviii, lxxi

Unintelligent Nursing, lxxiii

Vienna, Nurses in, xxi
Victoria Home for Nurses, xxix

Whitechapel Murders, the, vii
Wigan Infirmary, xxv
Women's Jubilee Offering, xxxiii
Wages and Washing, xxxv, xxxix, xliii, xlvii, liii, lvi
Women's Hospital at Redruth, xxxviii
Worcester General Infirmary, lv
Women as Druggists, lv
Women's Wages, lxxiii
Wedding, a Unique, lxxxiii
Worcester City Institution, ci
Wages or Charity, lxxix

Yellow Fever in Jacksonville and Florida, ii, v
York Nursing Institution, lxxxv, xcii

Zenana Missions, xix

### To Residents, Secretaries, and Matrons.

The Editor will be glad to have the Regulations and each Report as issued by Asylums, Hospitals, Convalescent and Nursing Institutions, as well as those of other Charities, and an early copy in duplicate of all Appeals and Festival Notices, etc., addressed to *The Editor, The Lodge, Porchester Square, London, W.* Any superintendent, secretary, matron, or other chief official who regularly sends such information may be registered, on application to the Publisher, to receive free of cost a cover for binding The Hospital in half-yearly volumes.

### Notice to Advertisers and Subscribers.

All Advertisements and Business Communications should be addressed to The Publisher, *not to the Editor*, The Hospital, 38, Old Jewry, E.C.

A Scale of Charges will be found in the Advertisement Sheets, page iii.

### IMPORTANT NOTICE.

THE FIRST CHAPTER OF

A NEW SERIAL STORY

ENTITLED

### FALSE IMPRESSIONS,

By CAROLINE FOTHERGILL,

*Author of "An Enthusiast," "A Voice in the Wilderness," &c.,*

Is commenced in the present issue, being No. 1 of Volume V.

Intending Subscribers should send their names to the publisher, 38, Old Jewry, London, E.C., without delay.

### NOTES AND NEWS.

### THE NATIONAL PENSION FUND FOR NURSES.

### Amusements and Relaxation.

### NEW PUZZLE PRIZE SCHEME.

COMMENCING OCTOBER 6th, ENDING DECEMBER 29th.

On October the 6th a New Prize Scheme will be commenced in these columns.

Prizes will be awarded for the best Original Puzzles in Prose or Verse, relating to any of the Social, Philanthropic, or Political topics of the day, and taking the form of Double Acrostics, Anagrams, Square Words, Decapitations, Transpositions, or any other Puzzles which the ingenuity of the competitors may suggest. (*Answers must accompany contributions.*)

*Readers* are requested to send in weekly the Number of Marks they consider each composition to be entitled to, three being the highest score for any one contribution.

Eight Prizes of Standard Books will be given, the choice of the book to be left to the winner.

First, Second, Third, Fourth, and Fifth Prizes for best Original Puzzles, of £1 1s., 15s., 10s. 6d., 7s. 6d, and 5s. each.

First, Second, and Third Prize for Solutions of 15s., 10s. 6d., and 5s. each.

N.B.—All contributions must reach the office not later than the *First Post* on *Thursday* in each week.

### THE CHILDREN'S CORNER.

During the past year we have offered the following

PRIZES
FOR MOST CORRECT ANSWERS TO PUZZLES.
One Pound a Month.
A PRIZE OF THREE GUINEAS
to the Competitor who shall have sent in the largest number of correct or best answers during the twelve months.

*In future these competitions will be discontinned.*

### Results of Fourth Week Puzzles.

No. 13. Double Acrostic —

B ismarc K

Y ar E

R omol A

O liphan T

N ollekon S

No. 14. Charade.—Toa t—Rack = Toastrack.

No. 15. Numerical Enigma.—Palmerston.

No. 16. Decapitation.—Dram, Ram, Am.

| Marks. | Sept. 22 d. | Total First, Second, Third, and Fourth Weeks. |
|---|---|---|
| A. C. K. | 4 | 15 |
| Gem | 4 | 14 |
| G. M. M. | — | 8 |
| Scrooge | 4 | 11 |
| Thanet | 4 | 15 |
| Tynesider | — | 3 |
| Judy | 3 | 9 |
| Primrose | 4 | 15 |
| J. W. M. | 4 | 12 |
| Agamemnon | 3 | 15 |
| G. E. R. | 4 | 14 |
| Happy Eliza | 3 | 9 |
| Penelope | 3 | 11 |
| Boadicea | 4 | 14 |
| Ikye Mo | 3 | 5 |
| Poodle | — | 2 |

*Next week we shall publish with the answers to Fifth Week Puzzles the names of the winners of the September and Yearly Prizes.*

### Conditions.

I. Every paper sent in must be clearly written, and one side only must be used. All competitors must mark at the head of their papers the number of their competition.

II. Each paper must be signed by the author with his or her real name and address. A *nom de plume* may be added if the writer does not desire to be referred to by us by his real name. In the case of all prizewinners, however, the real name and address will be published.

III. All communications relating to prize competitions should be addressed to "The Prize Editor, The Hospital, 38, Old Jewry, London, E.C." Competitors are requested not to include in letters, etc., to the Prize Editor, communications on matters of other business. All letters must be fully prepaid.

IV. The Prize Editor of The Hospital is the sole judge of the competitions, and his decision is in all cases final and without appeal.

V. The Prize Editor reserves the right to publish any paper sent in, whether it receives a prize or not. He does not hold himself responsible for any MSS. sent, nor can he undertake to return rejected competitions.

VI.—All children resident at school are requested to forward their *home* address in addition to the school one, when entering for the "Children's Corner" Competitions.

